# Intraoperative use of the machine learning-derived nociception level monitor results in less pain in the first 90 min after surgery

**DOI:** 10.3389/fpain.2022.1086862

**Published:** 2023-01-09

**Authors:** Imeen van der Wal, Fleur Meijer, Rivka Fuica, Zmira Silman, Martijn Boon, Chris Martini, Monique van Velzen, Albert Dahan, Marieke Niesters, Yaacov Gozal

**Affiliations:** ^1^ Department of Anesthesiology, Leiden University Medical Center, Leiden, Netherlands; ^2^ Department of Anesthesiology, Perioperative Medicine and Pain Treatment, Shaare Zedek Medical Center, Jerusalem, Israel; ^3^ Independent Biostatistician Consultant, Netanya, Israel

**Keywords:** postoperative pain, artificial intelligence, nociception monitoring during surgery, nociception, pain, monitoring

## Abstract

In this pooled analysis of two randomized clinical trials, intraoperative opioid dosing based on the nociception level-index produced less pain compared to standard care with a difference in pain scores in the post-anesthesia care unit of 1.5 (95% CI 0.8–2.2) points on an 11-point scale. The proportion of patients with severe pain was lower by 70%. Severe postoperative pain remains a significant problem and associates with several adverse outcomes. Here, we determined whether the application of a monitor that detects intraoperative nociceptive events, based on machine learning technology, and treatment of such events reduces pain scores in the post-anesthesia care unit (PACU). To that end, we performed a pooled analysis of two trials in adult patients, undergoing elective major abdominal surgery, on the effect of intraoperative nociception level monitor (NOL)-guided fentanyl dosing on PACU pain was performed. Patients received NOL-guided fentanyl dosing or standard care (fentanyl dosing based on hemodynamic parameters). Goal of the intervention was to keep NOL at values that indicated absence of nociception. The primary endpoint of the study was the median pain score obtained in the first 90 min in the PACU. Pain scores were collected at 15 min intervals on an 11-point Likert scale. Data from 125 patients (55 men, 70 women, age range 21–86 years) were analyzed. Sixty-one patients received NOL-guided fentanyl dosing and 64 standard care. Median PACU pain score was 1.5 points (0.8–2.2) lower in the NOL group compared to the standard care; the proportion of patients with severe pain was 70% lower in the NOL group (*p* = 0.045). The only significant factor associated with increased odds for severe pain was the standard of care compared to NOL treatment (OR 6.0, 95% CI 1.4 −25.9, *p* = 0.017). The use of a machine learning-based technology to guide opioid dosing during major abdominal surgery resulted in reduced PACU pain scores with less patients in severe pain.

## Introduction

Improvement of postoperative pain remains a challenging task for all involved in surgical patient care. A large number of patients still experiences moderate to severe postoperative pain despite the use of several analgesic techniques including multimodal pharmacological protocols, neuraxis and nerve blocks and various nonpharmacological interventions (*e.g.,* music therapy, cold packs, distraction) (1–3). In addition to causing patient distress and anxiety, postoperative pain is associated with delayed discharge, increased morbidity, persistent pain and prolonged consumption of opioids (4–7). One approach to improve postoperative pain scores may be to modify anesthetic practice, *i.e.,* to dose analgesic medication based on the nociceptive state of the patient rather than by using a fixed protocol based on hemodynamic measurements. In other words, we postulate that personalized management of nociceptive events during surgery may associate with improved postoperative pain scores, particularly in the post-anesthesia care unit (PACU). To this end, a novel monitor, the Nociception level (NOL), was developed with machine learning technology, that reliably tracks the patient nociceptive state and prompts analgesic dosing when the objective measure of nociception is high (8–12). We define nociception during surgery as “the central modulation of stimuli from surgical tissue damage into behavioral, autonomic and hormonal responses” (9). Note that the behavioral component of nociception (*e.g*., movement or a withdrawal response) is not detected during general anesthesia, particularly not when muscle relaxants are administered. Hormonal responses (see for example Figure 4 in Ref 12). may be measured in blood but are often only available at later times. Hence, the autonomic response us used to detect heightened nociception during clinical practice, however its sensitivity and specificity is often not optimal (9, 10).

The NOL is a nonlinear multiparameter that measures nociception from the following parameters: heart rate, heart rate variability amplitude of the finger photoplethysmogram, skin conductance level and their time derivatives, with greater sensitivity and specificity than either parameter alone (8–10). Random forest analysis was used to create the NOL index. This machine learning technique uses the combination of multiple variables of different origin to discover their intricate nonlinear linkages without the need for a description of a stochastic data model. The NOL scale has a range from 0 to 100, *i.e.,* from no nociception to extreme nociception. Validation studies showed with confidence that a NOL value of 25 distinguishes between non-nociceptive (NOL < 25) and nociceptive events (NOL > 25) (9–11). Therefore, the observation of NOL values that are greater than 25 (for at least 1 min) requires treatment with an analgesic drug such as an opioid, while values that are below 25 necessitate either no action or the reduction of analgesic medication that is administered continuously. Treatment is then independent of measured blood pressure and heart rate.

To strengthen our knowledge on the relationship between NOL-guided analgesic dosing during surgery and postoperative pain scores, we conducted a pooled analysis of two independent, randomized, controlled trials that compared the influence of intraoperative NOL-guided fentanyl to standard of care (SOC) on postoperative pain (12, 13). The two studies were equivalent with respect to study protocol and had common efficacy measures (Supplementary Digital Table S1). The results of both studies were that NOL-guided fentanyl dosing during surgery reduces pain scores in the post-anesthesia care unit (PACU) by 1.4–1.5 pints on an 11-point pain scale (from 0 = no pain to 10 = most severe pain imaginable). While the two studies had an identical design they evaluated 50 patients with predominantly surgical patients in the first study (SOLAR) (12), and 75 patient with an almost equal distribution among three surgery types (surgical 33%, urology 30% and gynecology 37%) in the second one (AbdomiNol) (13). The pooling enables to evaluate the effect based on larger sample size with better representation of surgery type, enables us generalizing results to a wider context especially identifying the specific patient populations that benefit from NOL-guided analgesia and leading to improved power to detect whether postoperatively there is less pain following NOL-guided fentanyl dosing. Moreover, the enlarged sample size enables a multivariable model to identify the effect of NOL on severe pain adjusted for confounders (14), such as age, gender, BMI, Surgery type, ASA and Site and revealing the only factor significantly related is the NOL. Finally, the pooling of data allowed us to analyze the three pain cohorts: intense, moderate and sever pain. We contend that our strategy will eventually lead to an improvement of pain in the PACU and all of its sequelae.

## Methods

This is pooled analysis of two earlier conducted and published trials with a similar protocol, the SOLAR trial and the Abdomi-Nol trial (12, 13). Both studies were prospective, double-blind (the patients and nurses who scored and treated the pain were unaware of the intraoperative treatment), parallel, randomized controlled trials on intraoperative nociception monitoring-guided opioid administration with primary endpoint median pain score in the first 90 min in the PACU and were conducted independently. The Abdomi-Nol study was designed to be confirmatory to the SOLAR trial. The SOLAR trial was conducted at two sites, a tertiary university center and a secondary referral center, both in the Netherlands (12). The Abdomi-Nol study was performed in a tertiary center in Israel (13).

Both studies utilized the PMD-200 nociception monitor, manufactured by Medasense Biometrics Ltd. (Ramat Gan, Israel). The device integrates several physiological variables that are known indicators of sympathetic activity to provide a single index of nociception, the NOL index. The PMD-200 sensing unit consists of a finger probe with four distinct sensors: photoplethysmogram, galvanic skin response, accelerometer, and a thermistor. The information from the accelerometer and the thermistor are used as a guardrail to ensure the algorithm performance but is not directly incorporated into the NOL calculation. Thousands of samples of these physiological variables (including heart rate, heart rate variability, vaso-constriction, and sweating) and their derivatives were recorded during major surgery of adult anesthetized patients and were annotated by expert clinicians for stimuli intensity and level of analgesia. These data were then used to train a random forest machine learning model, which is at the heart of the NOL algorithm. Although the model is locked, the algorithm “personalizes” its nociception index to the individual patient by implementing an adaptive weighting mechanism between the static model and the patient's unique physiologic responses during the surgical procedure. As the case progresses, the weighting of the patient's unique physiological response increases and the NOL output is adjusted accordingly. Separate datasets were used by the manufacturer to train, test and validate the NOL index (11).

In both studies, the NOL monitor finger clip was connected to the patient on the left or right middle finger before induction. In case of NOL-guided fentanyl dosing, the monitor screen was visible to the anesthesia providers. In case of SOC, the screen of the monitor was concealed. Pain scores in the PACU were obtained at 15 min intervals and intravenous doses of opioids were given according to local protocol until pain scores were considered acceptable (pain scores measured on a numerical rating scale, NRS, ranging from 0, no pain to 10, most imaginable pain), *i.e.,* NRS < 4. In both studies SOC was identical and was performed according to widespread clinical practice. In brief, but see for details below, fentanyl was given preemptively, prior to induction, followed by dosing base don the patient's condition and course of surgery, preferably isn such a way that hypertension and tachycardia were prevented. Still, in case of such hemodynamic instabilities further fentanyl was administered.

## Study design

***SOLAR study*** (12). After approval of the study by the local medical ethics committee the study was conducted at Leiden University Medical Center and Alrijne Hospital, Leiderdorp, both in the Netherlands. All protocol specifics, including inclusion and exclusion criteria can be found in the original paper (12) and in Supplementary Digital Table S1. The study is registered at https://trialsearch.who.int, under identifier NL7845. All patients gave written informed consent prior to enrolment. The study was conducted by anesthesiologists and residents that were trained in the use of the NOL monitor. Adult patients with ASA class 1-III scheduled to undergo elective laparoscopic or robot-assisted abdominal surgery without epidural anesthesia, local blocks or infiltration, were recruited. The patient, surgical team and PACU nurses were not informed on the patient allocation.

As stated above, in both groups preemptive fentanyl was given prior to intubation followed by dosing to preemptively prevent hemodynamic instabilities. The only difference between the NOL-guided and SOC groups was the trigger to administer additional fentanyl. In the test group, fentanyl dosing was dependent on the NOL-index, but blood pressure and heart rate were considered as well. In case the NOL index >25 for at least 60 s, 50–100 *μ*g fentanyl was administered in a patient >70 kg, and 25–50 *μ*g in a patient of 70 kg or less. Higher or lower fentanyl doses could be given or opioids could be given below the NOL threshold if felt needed by the attending anesthesiologist or resident. In case the index decreased below 25, no fentanyl was further administered. In the SOC group, fentanyl dosing was dependent on the course of surgery and on blood pressure and heart rate (NOL-index values were not available). This was left to the discretion of anesthesia care giver and based on local protocol.

***Abdomi-Nol study*** (13). This study was performed at the Shaare Zedek Medical Center, Jerusalem, Israel, after approval was obtained from the local medical ethics committee. Protocol details can be found elsewhere (13) and Supplementary Digital Table S1. The study was registered at www.clinicaltrials.gov under identifier NCT03970291. All patients gave written informed consent prior to enrolment. The study was conducted by anesthesiologists trained in the use of the NOL monitor. Adult ASA I-III patients scheduled to undergo elective laparoscopic abdominal, urologic or gynecologic procedures under general anesthesia without a planned epidural or local block were eligible for inclusion.

In the NOL-guided fentanyl dosing group, 0.5 µg/kg intravenous fentanyl was administered when NOL values were above 25 for at least 60 s. Higher or lower fentanyl doses could be given or opioids if felt needed by the anesthesiologist. In the SOC group, fentanyl dosing was at the discretion of the anesthesiologist and based on hemodynamic variables and course of surgery (see also above).

## Primary endpoint

The primary endpoint of both studies was the NRS for pain obtained by the PACU nursing staff in the first 90 min in the PACU. NRS < 4 was considered mild and acceptable, NRS from 4 to <7 moderate pain and 7 or higher severe pain. Pain scores of 4 or greater were treated in the PACU using a multimodal approach consisting of acetaminophen and/or an opioid. In the analyses, we highlight severe pain and maximal pain scores, as we consider these most agonizing and harmful to the patient.

## Statistical analyses

Prior to data pooling a comparison of general patient's characteristics between the 2 studies was conducted and there were no significant differences between the studies, except for surgery type distribution (Supplementary Table S2). Additionally, a comparison of NRS levels during PACU between 2 study arms demonstrated that groups were comparable with no significant differences between studies at all time points (NRS comparison between sites by Mann-Whitney *U* tests: *p* > 0.05 at all times points). The distribution of continuous variables was assessed using Shapiro & Wilk test. Continuous variables with non-normal distributions were expressed as median and interquartile range. Categorical variables were expressed as number and percentage. Comparisons of continuous variables between groups were performed with the Mann-Whitney U test for nonparametric variables, and the Fisher's exact-test or *χ*^2^-test for categorical variables. A logistic regression model was used to identify factors related to severe pain. Generalized linear models with the cluster bootstrap were applied to evaluate the difference in NRS accounting for the repeated measurement for each subject during the 90 min in PACU. This model was also used to evaluate the differences in specific subgroups. Pearson correlation coefficient were calculated for opioid dose during surgery vs. NRS in the PACU. *P*-values <0.05 were considered significant. Data were analyzed in R-4.0.3 (R Foundation for Statistical Computing, Vienna, Austria), SPSS Statistics (v-28.0, IBM, Armonk, NY, United States, or GraphPad Prism v-9.4.1 for macOS (GarphPad Software, San Diego, CA, United States). The raw data included in the study are available from the authors after agreement on purpose and protocol.

## Results

The study protocols (Supplementary Table S1), enrolled patient characteristics (Supplementary Digital Table S2) and pain scores in the PACU (Supplementary Digital Figure S1) from the two independent studies were sufficiently similar to allow a pooled analysis of the effect of the intervention (NOL-guided fentanyl dosing vs. SOC) on pain scores in the PACU. Hundred-twenty-five patients of either sex were enrolled in the studies (Table 1), with age range 21–86 years. The majority of patients were ASA class 2 (64%), with equal number of patients in ASA class 1 or 3 (18%). The types of surgeries were divided among three specialties: surgery (all abdominal cases) 47%, gynecology 33% and urology 20%. The two intervention arms were well balanced with respect to demographics, ASA classification and distribution of surgical procedures (Table 1).

**Table 1:** Patient characteristics of the subjects enrolled in the NOL-guided and the SOC groups.

	NOL-guided fentanyl dosing group (*n* = 61)	Standard of Care group (*n* = 64)	Total (*n* = 125)	*p*-value
**Sex**				
Male, No. (%)	22 (36)	33 (52)	55 (44)	0.081
Female, No. (%)	39 (54)	31 (48)	70 (56)	
**Age**				
Me dian (IQR), year	61 (49–67)	60 (43–70)	60 (45–69)	0.778
Range, year	(21–84)	(21–86)	(21–86)	
**BMI**				
Median (IQR), kg/m^2^	26 (22–30)	25 (24–29)	26 (23–29)	0.880
Range, kg/m^2^	(18–48)	(20–41)	(18–48)	
**Type of surgery**				
Urology, No. (%)	9 (15)	16 (25)	25 (20)	0.356
Gynecology, No. (%)	21 (34)	20 (31)	41 (33)	
Surgery, No. (%)	31 (51)	28 (44)	59 (47)	
**ASA**				
1, No. (%)	10 (17)	13 (20)	23 (18)	0.662
2, No. (%)	38 (62)	41 (64)	79 (64)	
3, No. (%)	13 (21)	10 (16)	23 (18)	

IQR, interquartile range; BMI, body mass index; ASA, american society of anesthesiologists.

**Figure 1:**
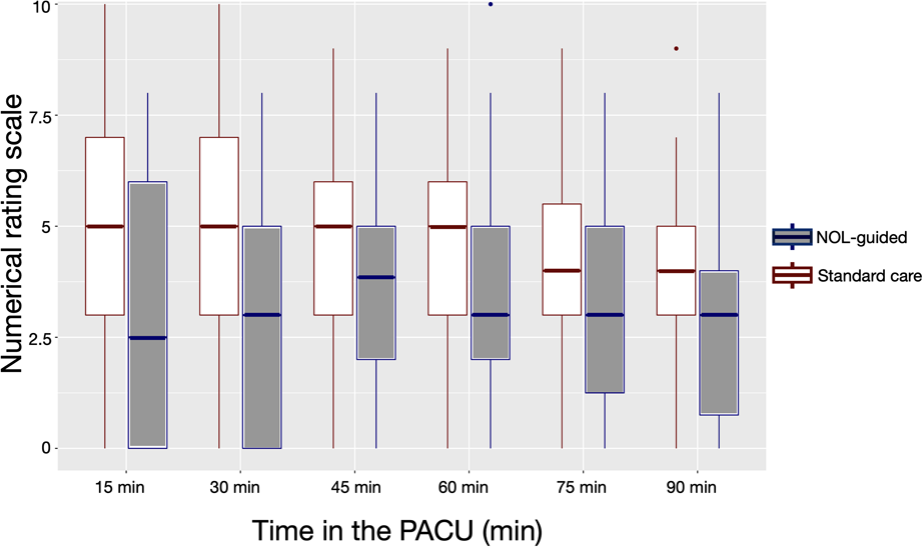
Boxplots of the effect of intraoperative nociception level (NOL)-guided fentanyl dosing and standard care (SOC) on postoperative pain scores.

The primary endpoint, postoperative pain during the first 90 min in the PACU, is presented in Figure 1. The figure demonstrates lower median NRS values at each time point in the NOL-guided group compared to SOC. With adjustments for time, sex, age and study site (Israel or the Netherlands), the two treatment groups differed significantly with median lower pain scores in the NOL-guided group compared to standard of care by 1.4 NRS points (95% CI 0.6–2.2), an effect that increased to 1.5 (0.8–2.2) NRS points after further adjustment for surgery type. The number of patients requiring no pain treatment increased from 10% (standard care) to 33% (NOL treatment; *p* = 0.002).

To identify specific patient populations that benefit from NOL-guided analgesia, generalized linear models with the cluster bootstrap were applied for each subgroup. Subgroups with the lower limit of the 95% confidence interval > 0 were: females (actual difference 1.9, 95% CI 1.0–3.0), patients ≤ 65 years (actual difference 1.8, 0.9- 2.9), ASA 1 patients (actual difference 2.0, 0.4–3.5), patients with a body mass index < 25 kg/m^2^ (actual difference 1.8, 0.6–3.0) or body mass index > 30 kg/m^2^ (actual difference 2.1, 0.5–3.7), patients undergoing urological surgery (actual difference 2.5, 1.2–3.7) and patients undergoing abdominal surgery (actual difference 1.4, 0.5–2.4).

**Figure 2:**
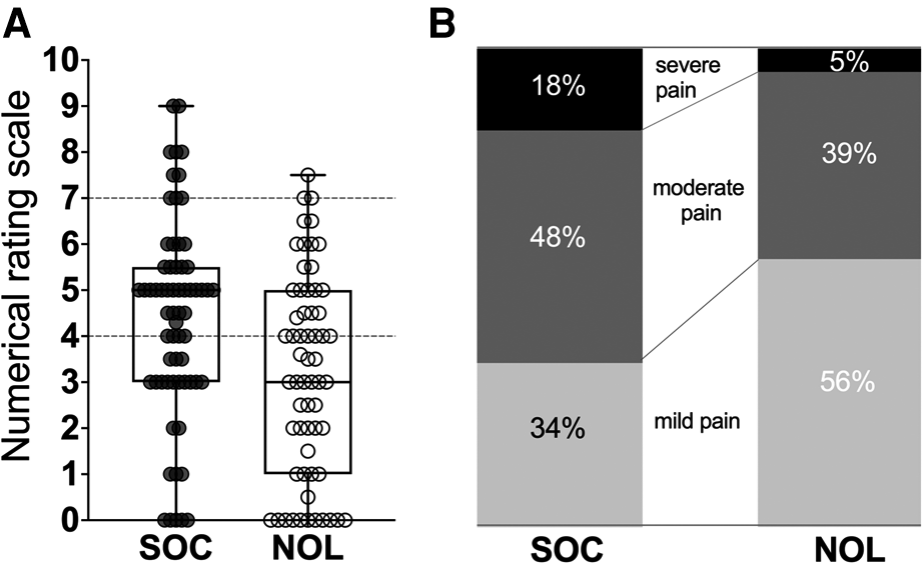
(**A**). Boxplot of the individual median pain scores observed during the patients’ stay in the PACU. (**B**). Percentage of patients with mild pain (NRS < 4), moderate pain (NRS > 4 and <7) and severe pain (NRS 7 or greater). SOC standard care, NOL Nociception Level-guided fentanyl dosing.

**Figure 3:**
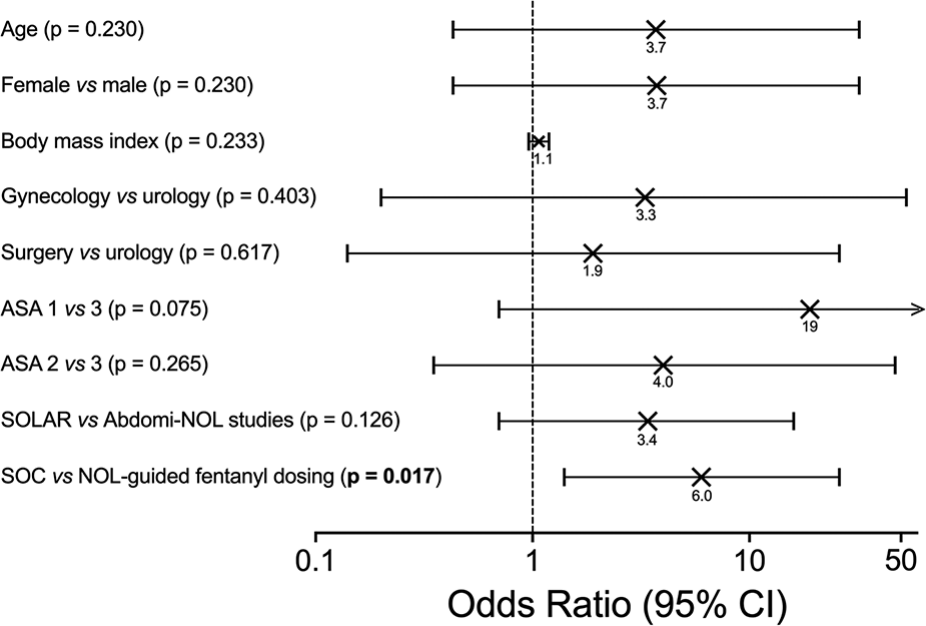
Logistic regression analysis identifying factors related to severe pain. The only significant factor associated with increased odds for severe pain was the standard of care approach for intraoperative fentanyl dosing compared to NOL-guided dosing.

The highest pain scores observed at any time throughout the 90 min stay in the PACU were 4.6 (NOL-guided group) and 6.2 (SOC; mean values with actual difference 1.7, *p* = 0.001) with 66% of patients in the NOL-guided group that had pains scores < 4 throughout their stay in the PACU vs. 10% in the SOC group. The number of patients with severe pain (NRS ≥ 7) was 11 in the SOC group and 3 in the NOL-guided group, *p* = 0.045 (Figure 2). Logistic regression identified the factors that were related to severe pain. The only significant factor associated with increased odds for severe pain of all factors considered (Figure 3) was the standard of care approach for intraoperative fentanyl dosing compared to NOL-guided dosing (OR 6.0 with 95% CI 1.4 to 25.9, *p* = 0.017). None of the other factors reached the level of significance.

**Figure 4:**
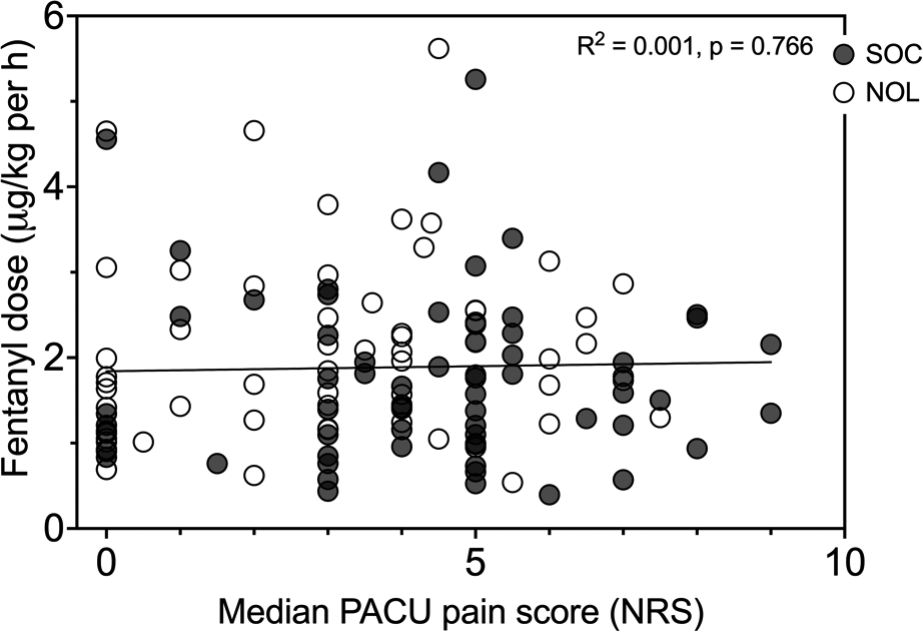
Intraoperative fentanyl dose vs. median pain score in the PACU for Nociception Level (NOL)-guided patients (open symbols) and standard of care patients (SOC, closed symbols). Each dot is one patient. The line is the linear regression curve of the full data set. Pearson correlation: complete data set *r*^2^ = 0.001, *p* = 0.766, NOL-guided patients *r*^2^ = 0.022, *p* = 0.246, and SOC patients *r*^2^ = 0.000, *p* = 0.891.

In Figure 4, the fentanyl consumption during surgery is plotted against the median pain scores in the PACU for all 125 patients. Analysis showed absence of correlation between opioid dosing and NRS.

## Discussion

Appropriate prevention of high postoperative pain scores remains challenging and all available effective techniques should be utilized to prevent development of pain-related complications. These complications can range from anxiety and distress to prolonged hospital stay, unplanned 30-day readmission and the chronification of pain (4–7). Equally relevant is the observation that in some European countries but also in the US, patients are discharged with an opioid prescription for treatment of ongoing pain as a result of shorter hospital stays (15, 16). Excessive prescribing of opioids for pain treatment after surgery has been identified as a public health problem and a potential contributor to patterns of opioid abuse and related harm (15).

In this publication, we present data on the use of a technology based on machine learning, the NOL monitor, to detect nociceptive events during surgery and treat them appropriately in order to reduce intraoperative nociception and prevent high pain scores in the PACU. In the pooled analysis of two controlled trials, we observed that titration of the fentanyl upon an intraoperative observation of an excessive nociceptive event resulted in significantly less PACU pain compared to the standard care with opioid dosing based on intermittent hemodynamic measurements. PACU pain scores was reduced in the NOL-treated group by 1.5 NRS points or 30%, a clinically meaningful result (17–20). NOL-treatment reduced median highest pain scores in the PACU (from 6.2 to 4.5 NRS points) and the proportion of patients with severe pain by 70% (from *n* = 11 patients to 3 patients; Figure 2). Despite multimodal pharmacotherapy, 17% of SOC patients suffered severe pain during their PACU stay; this number was reduced to 5% in patients who received intraoperative NOL-guided opioid dosing. This again is a significant observation and clinically relevant. Data from Cepeda et al. (17) indicate that a clinically meaningful improvement in pain scores is more challenging to attain in patients with severe pain than in patients with moderate pain.

Interestingly, the largest benefit of NOL-guidance was demonstrated in patients undergoing urological surgery and patients with a body mass index >30 kg/m^2^. Both of these groups had an difference in median PACU pain scores across treatment arms of more than 2 NRS points. Since a considerable proportion of patients in current clinical practice have a high body mass index, the value of using the NOL in particularly this population is of high clinical relevance.

In the NOL-guided group, fewer patients in the PACU experienced severe pain (Figure 2). Similar observations were made for the maximal pains scores at any time in the PACU. If we focus on the pain scores that trigger pharmacological treatment in our medical centers (*i.e.,* pain score of 4 NRS points or higher), we observed that intraoperative NOL-guided analgesia reduced the proportion of patients with pain scores ≥ 4 (at any time in the PACU) from 90% in SOC patients to 66% following NOL-guided analgesia. This means that while 90% of SOC patients required a treatment for their pain postoperatively, this was true for just two-thirds of the NOL-guided patients; in other words, one-third of the NOL-guided patients did not require any opioid or any other pain medication in the PACU. Logistic regression analysis (Figure 3) revealed further that intraoperative NOL-guided analgesia was the only variable that lowered the likelihood of experiencing severe pain in the PACU. These findings imply that disparities between groups in the number of patients reporting severe pain in the PACU are unrelated to patient or procedural variables.

One could reason that patients with severe pain in either treatment group received insufficient doses of fentanyl during surgery or that patients with mild or moderate pain were relatively overdosed. We determined, however, that pain scores were independent of fentanyl dose by plotting fentanyl consumption during surgery against the median pain scores in the PACU (Figure 4). This is an important observation and indicates that other factors than the magnitude of total fentanyl dose are responsible for the disparate outcomes of the two treatments. One such factor is likely the timing of fentanyl administration. We argue that when fentanyl was administered in response to a nociceptive event rather than triggered by an increase in blood pressure, the patient's nociceptive state was reduced throughout the surgical procedure, effectively resulting in less postoperative pain.

Combining individual data analysis from studies conducted at different sites into a pooled analysis requires uniformity in the patient population, surgical procedures, analgesic protocols, intervention and data collection (14, 21). Since the Abdomi-Nol study was a replica of the SOLAR study to some extent and designed to independently corroborate the results of the SOLAR study, the two studies were sufficiently similar to permit data pooling. Still, there were some differences between studies, such as the use of a monitor to control anesthetic depth in the SOLAR study, while dependence on end-tidal volatile gas concentrations in the Abdomi-Nol study. Nevertheless, the two approaches are sufficiently comparable that they did not impact our current results. Nonetheless, pain sensitivity and attitudes toward pain scoring, may have been different in ethnically divergent Dutch and Israeli patient populations, despite the use of identical metrics (22, 23). The comparable pain scores in the PACU between the two sites (Supplementary Digital Figure S1) imply that such differences were minor.

In conclusion, intraoperative machine-learning based NOL-guided dosing of fentanyl as opposed to dosing fentanyl based on blood pressure and heart rate, resulted in (1) improved PACU pain scores, (2) fewer patients with severe pain, (3) a greater proportion of patients who did not require any opioid treatment in the PACU compared to standard care; lastly, (4) our analysis showed that the predictor of less severe pain in the PACU was NOL-guided fentanyl dosing. These finding are pertinent and may aid in minimizing the prevalence of severe pain after surgery and all of its negative repercussions.

Relating to this last remark, it is important to reflect on the consequences of less PACU pain and a reduced number of patients that required opioid treatment in the PACU in light of the current opioid crisis. One of the causes of the opioid crisis, at least in the Netherlands, is the fact that hospital stay is currently relatively short and many patients are discharged from the hospital, while still in pain, with an opioid prescription (16). Although our study was not designed to study the long-term effects of less PACU pain and reduced PACU opioid requirements, we argue that this will assist in reducing long-term opioid consumption both in-house and possibly even after discharge. Further studies are needed to address this issue.

## Data Availability

The data are available from the authors after agreement has been obtained regarding purpose of analysis and protocol.
