# Supplementary material for: Intraoperative use of the machine learning-derived nociception level monitor results in less pain in the first 90 min after surgery
Source: Front Pain Res (Lausanne). 2023 Jan 9;3:1086862. doi: 10.3389/fpain.2022.1086862 (PMC9869062; doi:10.3389/fpain.2022.1086862)

Supplemental Digital Figure 1. Median pain scores in the postanesthesia care unit (PACU) in the SOLAR and Abdomi-Nol studies and the combined data set. The data are from the complete population (NOL-guided and standard care groups). Pain scores were compared at each time point using a Mann-Whitney U test with p-values > 0.05 at all time points.

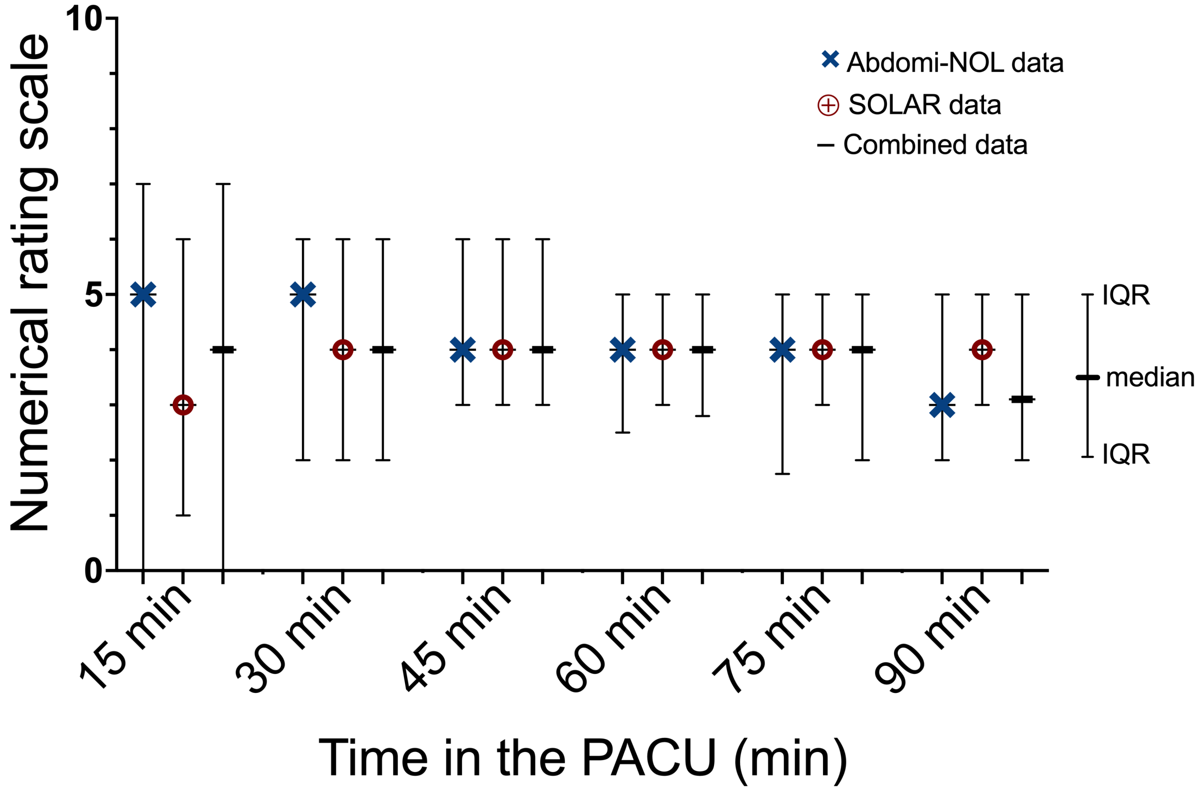

Supplement: Supplementary file 1 [file Datasheet1.pdf]
