# Supplementary material for: Intraoperative use of the machine learning-derived nociception level monitor results in less pain in the first 90 min after surgery
Source: Front Pain Res (Lausanne). 2023 Jan 9;3:1086862. doi: 10.3389/fpain.2022.1086862 (PMC9869062; doi:10.3389/fpain.2022.1086862)
Supplement: Supplementary file 2 [file Datasheet2.pdf]

Supplemental Digital Table 1. Comparison of study protocols of the SOLAR and Abdomi-NOL studies

	**SOLAR**	**Abdomi-Nol**
Study design	Two-center, prospective, randomized, parallel, double blind.	Single center, prospective, randomized, parallel, double blind
Number of patients	50 (22 men, 28 women)	75 (33 men, 42 women)
Primary endpoint	Postoperative pain scores during the first 90 min in the PACU.	Postoperative pain scores during the first 90 min in the PACU.
Inclusion criteria	• 18 years and older.<br>• ASA I-III.<br>• Elective major abdominal surgery.	• 18 years and older.<br>• ASA I-III.<br>• Elective major abdominal surgery.
Exclusion criteria	• Use of epidural, local or infiltration anesthesia/ analgesia.<br>• Pregnancy/lactation.<br>• BMI > 40 kg/m.<br>• Hypertension (systolic blood pressure > 160 mmHg) prior to induction.<br>• Hypotension (MAP < 60 mmHg) prior to induction.<br>• Heart rate < 45/min or > 90/min prior to induction.<br>• CNS disorder (incl. alcohol abuse, illicit drug use, opioid tolerance or use of psychoactive medication).	• Use of epidural or local anesthesia/ analgesia.<br>• Pregnancy/lactation.<br>• Any current or previous heart rate other than a sinus rhythm.<br>• CNS disorder (incl. alcohol abuse, illicit drug use, opioid tolerance or use of psychoactive medication) current or in the last 6 months.
Base protocol	• Induction with fentanyl, propofol and rocuronium.<br>• Maintenance with sevoflurane, fentanyl and rocuronium.<br>• Fentanyl dosing to preemptively prevent hemodynamic instability.<br>• Preemptive analgesia given 45-60 min prior to the end of surgery: IV acetaminophen 1 g and morphine 0.1-0.15 mg/kg or 0.2-0.3 mg/kg piritramide.<br>• Antiemetic, ondansetron, after induction.	• Induction with fentanyl, propofol and rocuronium.<br>• Maintenance with sevoflurane, fentanyl and rocuronium. Fentanyl dosing to preemptively prevent hemodynamic instability.<br>• Preemptive analgesia given 30-45 min prior to the end of surgery: IV acetaminophen 1 g and morphine 0.1-0.15 mg/kg.<br>• Antiemetic, ondansetron, prior to the end of surgery.
Depth of anesthesia monitoring	• Bispectral index of the electroencephalogram.	• Monitoring of end-tidal sevoflurane concentration.
Protocol in the NOL-guided fentanyl dosing group	• Goal: NOL < 25.<br>• NOL > 25 for at least 60 s: fentanyl bolus dose (50-100 μg in a patient ≥70 kg; 25-50 μg in a patient <70 kg); next evaluation after 5-10 min.	• Goal: NOL < 25.<br>• NOL > 25 for at least 60 s: fentanyl bolus dose (0.5 μg/kg); next evaluation after 5 min.

	• NOL < 25 and MAP > 100 mmHg: fentanyl bolus dose<br>• NOL > 25 and MAP < 60 mmHg: vasoconstrictor given.	• NOL < 25 and MAP > 100 mmHg: vasodilator.
Postoperative pain control	• According to local protocol with either morphine or piritramide.	• According to local protocol with either morphine or tramadol.
Data collection	• In Castor electronic data capture system (Castoredc)*	• In Castoredc
Randomization	• Within Castoredc	• Within Castoredc

MAP mean arterial pressure; CNS central nervous system; PACU post-anesthesia care unit; IV intravenous; ASA American Society of Anesthesiologists; * castoredc.com.
