# Supplementary material for: Intraoperative use of the machine learning-derived nociception level monitor results in less pain in the first 90 min after surgery
Source: Front Pain Res (Lausanne). 2023 Jan 9;3:1086862. doi: 10.3389/fpain.2022.1086862 (PMC9869062; doi:10.3389/fpain.2022.1086862)
Supplement: Supplementary file 3 [file Datasheet3.pdf]

Supplemental Digital Table 2. Comparison of patient characteristics between SOLAR and Abdomi-NOL studies

	SOLAR<br>(*n* = 50)	Abdomi-Nol<br>(*n* =75)	Total<br>(*n* =125)	p-value
**Sex**				
Male, No. (%)	22 (44)	33 (44)	55 (44)	1.000
Female, No. (%)	28 (56)	42 (56)	70 (56)	
**Age**				
Median (IQR), year	60 (44-67)	61 (47-70)	60 (45-69(	0.443
Range, year	22-83	21-86	21-86	
**BMI**				
Median (IQR), kg/m^2	26 (23-29)	25 (24-29)	26 (23-29)	0.621
Range, kg/m^2	19-42	18-48	18-48	
**Type of surgery**				
Urology, No. (%)	3 (6)	22 (30)	25 (20)	< 0.001
Gynecology, No. (%)	13 (26)	28 (37)	41 (33)	
Surgery, No. (%)	34 (68)	25 (33)	59 (47)	
**ASA**				
1, No. (%)	12 (24)	11 (15)	23 (18)	0.314
2, No. (%)	31 (62)	48 (64)	79 (63)	
3, No. (%)	7 (14)	16 (21)	23 (18)	

IQR interquartile range; BMI body mass index; ASA American Society of Anesthesiologists.
